# Supplementary material for: The Systems Biology Research Tool: evolvable open-source software
Source: BMC Syst Biol. 2008 Jun 29;2:55. doi: 10.1186/1752-0509-2-55 (PMC2446383; doi:10.1186/1752-0509-2-55)
Supplement: Additional file 1 — SBRT Archive. An archive of the current version of the Systems Biology Research Tool. [file 1752-0509-2-55-S1.zip › sbrt-1.4.0/doc/developers_guide/api/sbrt/kernel/math/algebra/class-use/Matrix.html]

Uses of Interface sbrt.kernel.math.algebra.Matrix


|  |  |  |
| --- | --- | --- |
| **Overview** \| **Package** \| **Class** \| **Use** \| **Tree** \| **Deprecated** \| **Index** \| **Help** | |  |
| PREV   NEXT | **FRAMES**    **NO FRAMES**     **All Classes** |  |


---


## **Uses of Interface sbrt.kernel.math.algebra.Matrix**

| Packages that use Matrix | |
| --- | --- |
| **sbrt.kernel.math.algebra** | Provides classes and interfaces for creating objects relevant to algebra. |

Uses of Matrix in sbrt.kernel.math.algebra

| Subinterfaces of Matrix in sbrt.kernel.math.algebra | |
| --- | --- |
| `interface` | `DoubleMatrix<R,C>`             This interface is used to represent matrices whose elements are strictly double precision numbers. |
| `interface` | `DoubleMatrixBuilder<R,C>`             This interface is used to represent mutable matrices whose elements are strictly double precision numbers. |
| `interface` | `MatrixBuilder<R,C,E>`             This interface is used to represent mutable matrices. |
| `interface` | `SmdMatrix<R,C>`             This interface is used to represent sparse mutable matrices containing strictly double precision numbers as elements. |
| `interface` | `SparseDoubleMatrix<R,C>`             This interface is used to represent sparse matrices whose elements are strictly double precision numbers. |

| Classes in sbrt.kernel.math.algebra that implement Matrix | |
| --- | --- |
| `class` | `AbstractMdMatrix<R,C>`             This abstract class is a skeleton implementation of `DoubleMatrixBuilder`. |
| `class` | `AbstractSmdMatrix<R,C>`             This abstract class is a skeleton implementation of `SmdMatrix`. |
| `class` | `DoubleMatrixWrap<R,C>`             This class is used to wrap mutable matrices as an immutable `DoubleMatrix`. |
| `class` | `HashMatrix<R,C>`             This class is a `java.util.LinkedHashMap` based implementation of `SmdMatrix`. |
| `class` | `MatrixWrap<R,C,E>`             This class is used to wrap mutable matrices as an immutable matrix. |
| `class` | `RowOrTreeMatrix<R extends Comparable<? super R>,C extends Comparable<? super C>>`             This class is a `java.util.TreeMap` based implementation of `SmdMatrix`. |
| `class` | `SparseDoubleMatrixWrap<R,C>`             This class is used to wrap mutable matrices as an immutable `SparseDoubleMatrix`. |
| `class` | `TreeMatrix<R extends Comparable<? super R>,C extends Comparable<? super C>>`             This class is a `java.util.TreeMap` based implementation of `SmdMatrix`. |

| Methods in sbrt.kernel.math.algebra that return Matrix | |
| --- | --- |
| `Matrix<R,C,E>` | `MatrixWrap.getColumn(java.lang.Object columnIndex)`             Returns the column corresponding to the provided column index. |
| `Matrix<R,C,E>` | `Matrix.getColumn(java.lang.Object columnIndex)`             Returns the column corresponding to the provided column index. |
| `Matrix<R,C,E>` | `MatrixWrap.getRow(java.lang.Object rowIndex)`             Returns the row corresponding to the provided row index. |
| `Matrix<R,C,E>` | `Matrix.getRow(java.lang.Object rowIndex)`             Returns the row corresponding to the provided row index. |

| Methods in sbrt.kernel.math.algebra with parameters of type Matrix | |
| --- | --- |
| `void` | `AbstractSmdMatrix.setElements(Matrix<? extends R,? extends C,? extends java.lang.Double> m)`             Sets the corresponding elements of this matrix to those of the provided matrix. |
| `void` | `MatrixBuilder.setElements(Matrix<? extends R,? extends C,? extends E> m)`             Sets the corresponding elements of this matrix to those of the provided matrix. |
| `<R,C,E> void` | `MatrixTransposer.transpose(Matrix<R,C,E> matrix, MatrixBuilder<? super C,? super R,E> emptyMatrix)`             Fills `emptyMatrix` with the transpose of `matrix`. |

Constructors in sbrt.kernel.math.algebra with parameters of type Matrix

`MatrixWrap(Matrix<R,C,E> m)`
  
          Constructs a new wrapper around the provided matrix.

---


|  |  |  |
| --- | --- | --- |
| **Overview** \| **Package** \| **Class** \| **Use** \| **Tree** \| **Deprecated** \| **Index** \| **Help** | |  |
| PREV   NEXT | **FRAMES**    **NO FRAMES**     **All Classes** |  |


---
